# A Polarization-Insensitive and Highly Sensitive THz Metamaterial Multi-Band Perfect Absorber

**DOI:** 10.3390/mi15111388

**Published:** 2024-11-16

**Authors:** Gang Tao, Qian Zhao, Qianju Song, Zao Yi, Yougen Yi, Qingdong Zeng

**Affiliations:** 1 School of Environment and Resource, Southwest University of Science and Technology, Mianyang 621010, China; 2 Tianfu Institute of Research and Innovation, Southwest University of Science and Technology, Chengdu 610218, China; 3 Joint Laboratory for Extreme Conditions Matter Properties, Southwest University of Science and Technology, Mianyang 621010, China; zq2452872471@163.com (Q.Z.); qjsong@swust.edu.cn (Q.S.); yizaomy@swust.edu.cn (Z.Y.); 4 School of Chemistry and Chemical Engineering, Jishou University, Jishou 416000, China; 5 College of Physics, Central South University, Changsha 410083, China; yougenyi@csu.edu.cn; 6 School of Physics and Electronic-information Engineering, Hubei Engineering University, Xiaogan 432000, China; zengqingdong2005@163.com

**Keywords:** THz, metamaterial, Dirac semimetals, refractive index sensitivity

## Abstract

In this article, we present a terahertz (THz) metamaterial absorber that blends two types of coordinated materials: Dirac semimetals and vanadium dioxide. Compared to other absorbers on the market, which are currently non-adjustable or have a single adjustment method, our absorber is superior because it has two coordinated modes with maximum adjustment ranges of 80.7% and 0.288 THz. The device contains four flawless absorption peaks (M1, M2, M3, and M4) spanning the frequency range of 2.0 THz to 6.0 THz, all with absorption rates greater than 99%. After calculation, the relative impedance of the device matches with that in free space, resulting in perfect absorption. In addition, our absorber has extremely excellent polarization insensitivity but is highly sensitive to changes in the environmental refractive index, with the highest environmental refractive index sensitivity of 716 GHz/RIU (gigahertz per refractive index unit). To sum up, the terahertz metamaterial absorber we showed has four perfect absorption peaks, high sensitivity, and stable polarization. This means it could be useful in areas like changing electromagnetic waves, making new sensors, and switching.

## 1. Introduction

The terahertz (THz) wave is the aggregation of all electromagnetic waves in the frequency range of 0.1 THz–10 THz [1,2,3]. Benefiting from its superior bandwidth conditions, it is predicted to be one of the best match frequency ranges for future 6G communication [4,5,6]. Further research on terahertz waves has found that they have great potential value in the aspects of electromagnetic communication [7], biomedical diagnosis [8], object non-destructive testing [9], and atmospheric environment monitoring [10]. However, the terahertz wave has low compatibility and poor response with traditional precious metal materials (such as gold, silver, copper, etc.) due to the limitation of photon energy within the THz frequency range. This greatly increases the inconvenience of the design of terahertz devices and restricts the innovation of terahertz-related technologies [11]. In order to overcome this obstacle, metamaterials have been included in the research of terahertz functional devices. Within the word metamaterials, the Latin root “meta” means “beyond, alternative,” while “material” refers to various materials that exist in nature. Just as the name implies, metamaterials refer to advanced composite materials created independently by humans that are not originally present in nature [12,13]. Their greatest feature is that they can show excellent physical and chemical properties that ordinary materials cannot possess. For instance, metamaterials have a negative index of refraction and negative permittivity and are also known as “left-handed materials” due to the law that the internal propagation of electric or magnetic fields conforms to the right-handed relationship observed during the dissemination of traditional materials [14,15]. Metamaterials are engineered to create devices capable of both manipulating and absorbing electromagnetic radiation. These metamaterials are typically composed of periodic metal structure arrays and lossy media. However, such metamaterial devices often suffer from limited flexibility and functionality due to the highly rigid and static properties of the metals used. Consequently, these devices are constrained to single-purpose applications unless their structural design is significantly altered. Recently, research on terahertz absorbers based on coordinated material Dirac semimetal design has been very active. Dirac semimetal (DSM) is a quantum-state material that is a three-dimensional representation of graphene. Its valence band and conduction band overlap at the Dirac point, belonging to a semimetal with extremely low inherent losses and possessing high carrier mobility similar to or even far exceeding that of graphene. In 2015, Kharzeev et al. discovered that the metal response of Dirac semimetals is linked to the presence of surface plasmon polaritons (SPPs), indicating that Dirac semimetals are an excellent loss material significant for designing metamaterial absorbers [16]. In addition, the dielectric constant of DSM can be regulated by chemical doping and applying gate voltage to its Fermi energy [17]. In 2019, the team of Meng and Que proposed a three-band terahertz metamaterial absorber based on bulk Dirac semimetals [18]. The simulation results indicated that their absorber has a perfect absorption rate of over 99% for all three absorption peaks in TE (transverse electric) and TM (transverse magnetic) modes. Above all things, when they adjusted the Fermi energy of the top-level DSM, all absorption peaks shifted. Additionally, the absorption rate could remain at the same extremely high level as before, while the resonant frequency increased. Their design demonstrates the rationality of the practical application of high-performance terahertz band absorption systems based on DSM materials. Vanadium dioxide (VO_2_) is a typical phase change material with a phase transition temperature close to 340 K [19,20]. The phase transition process involves a coupled “metal-insulator” transition and changes in the crystal structure, reflected in variations in physical properties like conductivity and the refractive index, as well as transformations between monoclinic and tetragonal forms [21]. Both the absorbers proposed by Meng and Zuo have a drawback, being the single-tunable method and limited device functionality. So, we envisioned the possibility of combining DSM and VO_2_ to design a terahertz device with multiple coordination modes.

In this article, we designed a terahertz device by combining two compatible materials. Our device has a three-layer structure, with DSM and VO_2_ serving as the top absorption layer and bottom substrate, respectively, forming a stable Fabry Perot cavity. The resonance mode excited by the Fabry Perot cavity provides perfect absorption for the device, with four perfect absorption peaks in the frequency range of 2.0 THz to 6.0 THz. We adjusted the relevant functions of the device by controlling the temperature and changing the bias voltage, namely through triggering the VO_2_ phase transition and adjusting the Fermi level of DSM, thereby changing the peak value and position of the four absorption peaks. The maximum adjustment range of the former is 80%, while the latter is 0.288 THz. The structure’s strong symmetry allows our absorber to maintain good polarization stability while significantly decreasing the device’s preparation time and cost. Therefore, the absorber we proposed has potential application value in photon detection, electromagnetic stealth, and advanced terahertz devices. Additionally, our absorber’s high sensitivity to variations in the refractive index allows it to detect substances effectively thanks to its special terahertz characteristics. As a result, it exhibits significant promise for sensing applications, particularly in chemical and biological analytes.

## 2. Design and Materials

### 2.1. Structure Mode

**Figure 1:**
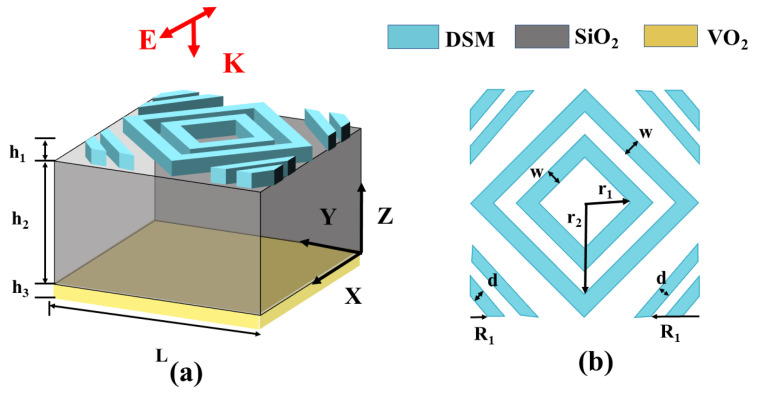
(**a**) Three-dimensional view of device structure. (**b**) DSM layer structure display.

As shown in Figure 1, our device has a three-layer structure consisting of a metal layer, a dielectric layer, and a metal layer. From bottom to top, the materials of each layer are as follows: vanadium dioxide (VO_2_), silicon dioxide (SiO_2_), and Dirac semimetal (DSM), with thicknesses of 1 μm, 18 μm, and 7 μm. The length of the vanadium dioxide substrate and silicon dioxide dielectric layer in both X and Y directions is L = 40 μm. The absorber contains two rhombus rings at the top center and two on each of the four diagonals for a total of eight trapezoidal structures. The DSM layer’s relevant structural parameters are listed below: w=4 μm, $r_{1}=8\;\mu m$, $r_{2}=14\;\mu m$. In simulation calculations, the initial values of the Fermi energy of DSM and the conductivity of VO_2_ were set to 90 Mev and 250,000 S/m, respectively [22,23]. In simulation calculations, the Fermi energy of DSM and the conductivity of VO_2_ were initialized to 90 Mev and 250,000 S/m, respectively. In further research on the device’s operation, we replicated DSM surface doping and VO_2_ phase transition by modifying the values of these two factors in order to evaluate its two controlling ways.

The structural design of our absorber focuses primarily on facilitating plasmonic resonance on the absorber’s surface and achieving effective coupling between its various components. First, at specific resonance frequencies, the metamaterial structure generates electromagnetic resonances, creating zones of intense electromagnetic field concentration, either on the surface or within the material. This resonance effectively “captures” the energy of incident THz waves in these regions, preventing the energy from escaping. In addition, we incorporated two annular structures in the design, enabling mutual electromagnetic field interactions that result in coupling effects. This coupling phenomenon induces a frequency splitting of the original resonance, dividing a single resonance frequency into multiple peaks, which produces multiple absorption bands.

The relevant design and simulation calculations of our device were completed in the professional electromagnetic simulation software Computer Simulation Technology 2020 (CST) [24,25]. The environmental background was set to air, and the boundary conditions of X, Y, and Z were sequentially set to unit, unit, and open (adding space). Using the finite element method, simulation calculations were completed using a frequency domain solver. In the actual production of this device, we put forward the following manufacturing process. First of all, the VO_2_ layer at the bottom can be deposited on a pure silicon substrate by using magnetron sputtering. The SiO_2_ in the middle is also deposited on the prepared VO_2_ layer using the same method. The preparation of the DSM absorption layer at the top requires two steps. The first step is to prepare a DSM layer with a thickness of 7 μm on the SiO_2_ dielectric layer by Molecular Beam Epitaxy (MBE). The second step is to remove excess parts through Deep Ultraviolet Lithography (DUV) and carving out specific shape structures [26,27]. In practical applications, THz metamaterial structures with various characteristics and frequency responses can be fabricated by precisely adjusting MBE process parameters, such as the deposition rate, the substrate temperature, and the material flow rate. For instance, an increased substrate temperature promotes atomic-level flatness in the film, thereby minimizing grain boundary defects and enhancing the absorber’s overall performance. Furthermore, leveraging the high-resolution capability of DUV lithography enables precise optimization of structural dimensions and geometry, thereby improving electromagnetic field localization and significantly enhancing THz wave absorption efficiency.

### 2.2. Optical Characterization of Metamaterials

In the terahertz domain, using Kubo formalism, the dynamic conductivity (σ) of DSM can be expressed as follows [28,29]:

(1) $$Re\sigma =\frac{e^{2}gk_{F}}{24\pi h}\left(\frac{h\omega }{E_{F}}+\frac{ih\tau^{-1}}{E_{F}}\right)\theta \left(\frac{h\omega }{E_{F}}+\frac{ih\tau^{-1}}{E_{F}}-2\right)$$

(2) $$Im\sigma =\frac{e^{2}gk_{F}}{24\pi h}\left[\frac{4}{\frac{h\omega }{E_{F}}+\frac{ih\tau^{-1}}{E_{F}}}-\frac{h\omega }{E_{F}}+\frac{ih\tau^{-1}}{E_{F}}\ln\left(\frac{4{\varepsilon_{c}}^{2}}{\left|{\left(\frac{h\omega }{E_{F}}+\frac{ih\tau^{-1}}{E_{F}}\right)}^{2}-4\right|}\right)\right]\;$$

In the above equation, *e*, h, and g are, respectively, the electron charge, the reduced Planck constant, and the degeneracy factor, where g=40. ω is the frequency and *E_F_* represents the Fermi energy. τ is the intrinsic time and $\tau =4.5\times 10^{-13}$. $k_{F}=E_{F}/\hbar \nu_{F}$ is the Fermi momentum, with $\nu_{F}=10^{6}m/s$ being the Fermi velocity. $\varepsilon_{c}=E_{c}/E_{F}=3$.

*θ*(*t*) is the Riemann–Siegel theta function *θ*(*t*), and its expression is as follows [30]:

(3) $$\theta \left(t\right)=-\frac{i}{2}\left[ln\Gamma \left(\frac{1}{4}+\frac{it}{2}\right)-ln\Gamma \left(\frac{1}{4}-\frac{it}{2}\right)\right]-\frac{tln\left(\pi \right)}{2}\;$$

**Figure 2:**
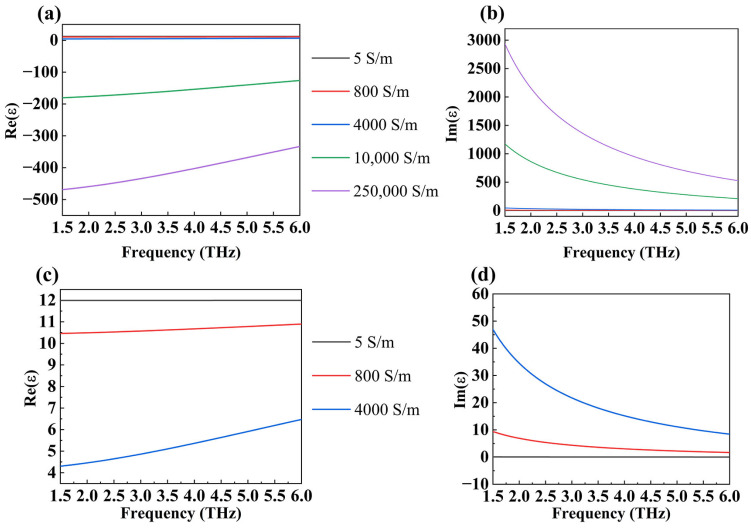
The real (**a**) and the imaginary (**b**) components of the permittivity for VO_2_ as a function of frequency. (**c**,**d**) is a zoomed in view of the overlapping area in the first two images.

If we take the interband electronic transitions and the dual band models into account, we can express the specific inductive capacity of DSM as follows [31]:

(4) $$\varepsilon =\varepsilon_{b}+i\frac{\sigma }{\varepsilon_{0}\omega }$$

where *ε*_0_ is the permittivity of the vacuum and *ε_b_* = 1. We plotted the functional relationship between the conductivity and frequency of DSM with different Fermi energies in the frequency range of 2.0 THz to 6.0 THz, as displayed in Figure 2. The optical properties of vanadium dioxide within the THz range can be characterized as follows [32]:

(5) $$\varepsilon_{VO_{2}}=\varepsilon_{\infty }-\frac{{\omega_{p2}}^{2}}{\omega (\omega +i\omega_{t2})}$$

In the above equation, $\varepsilon_{\infty }=12$ is the dielectric constant at an infinite frequency, and $\omega_{t2}=5.75\times 10^{15}S^{-1}$ is the collision frequency. ${\omega_{p2}}^{2}=\frac{\sigma }{\sigma_{0}}{\omega_{p0}}^{2}$ with $\omega_{p0}=1.45\times 10^{15}s^{-1}$ as the initial value of the plasma frequency for VO_2_ [33]. In our simulation calculations, we modeled the phase transition of VO_2_ by varying its electrical conductivity. Specifically, we considered five different conductivity values for VO_2_: 5 S/m, 800 S/m, 4000 S/m, 10,000 S/m, and 250,000 S/m. Additionally, we generated a diagram showing the variation of VO₂ conductivity with a frequency in the range of 1.5 to 6.0 THz, as depicted in Figure 2. For the purposes of this study, VO₂ is classified as non-metallic when its conductivity is 5 S/m and as metallic when its conductivity reaches 250,000 S/m. In optics, an object’s energy processing of light can be loosely split into three categories: absorption, reflection, and transmission. When light strikes an item, some of it is reflected by its surface (*R*), some enters the object (*T*), and the remainder is absorbed by the object (*A*). Because all light that strikes an item is processed by at least one of these three methods, the sum of these three methods should equal one. So, the relationship between the absorptivity, reflectivity, and transmissivity of an absorber can be expressed as follows: A=1−R−T. This relationship is based on the law of conservation of energy in physics. For our absorber, the thickness of the vanadium dioxide substrate far outweighs the skin depth, and the transmittance is almost 0. So, the absorptivity of the device can be written as follows: $A=1-R=1-{\left|S_{11}(\omega )\right|}^{2}$, where $S_{11}(\omega )$ is the reflection coefficient in the *S* parameter [34,35,36].

## 3. Results and Discussions

### 3.1. Absorption Performance

**Figure 3:**
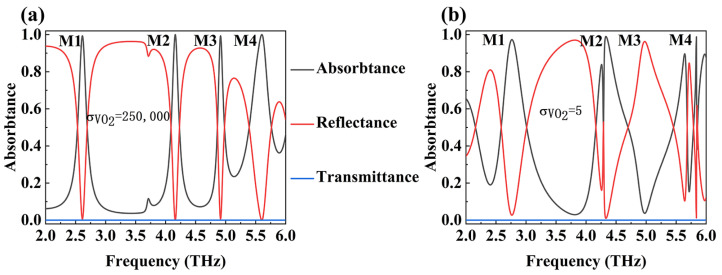
(**a**) The absorption spectrum of the device when the VO_2_ conductivity is 250,000 S/m, and (**b**) the absorption spectrum of the device when the VO_2_ conductivity is 5 S/m.

Figure 3 shows the absorbance spectra of the device when the conductivity of VO_2_ is equal to 250,000 S/m (metallic state) and 5 S/m (non-metallic state). As shown in Figure 3a, there are four different absorption modes (M1, M2, M3, and M4) in the frequency range of 2.0 THz to 6.0 THz when VO_2_ is in the metallic state. When VO_2_ is in a non-metallic state, the absorption rates of the four peaks are 58.8%, 48.4%, 10.2%, and 80.7%, respectively. When VO_2_ is in the metallic state, our absorber and the conventional metal–medium–metal Hamburg structure are the same, forming a stable cavity resonator. When the device is incident by THz, plasmon resonance is stimulated at the upper and lower interfaces of the internal SiO_2_ dielectric layer, causing electromagnetic waves to oscillate and couple inside the device, ultimately being absorbed by losses [37]. When VO_2_ becomes non-metallic, the previous cavity resonator structure loses the bottom metal reflection layer, greatly reducing the coupling with electromagnetic waves and undermining the device’s ability to absorb THz waves [38]. Additionally, VO_2_ undergoes a phase transition due to temperature changes, transforming from a metallic state to a non-metallic state. This transformation is reversible and does not affect the properties of VO_2_ itself. The results indicate that the absorption degree for terahertz waves of our device is to some extent adjustable, with a maximum adjustment range of 80%.

### 3.2. The Physical Mechanism of Absorption

**Figure 4:**
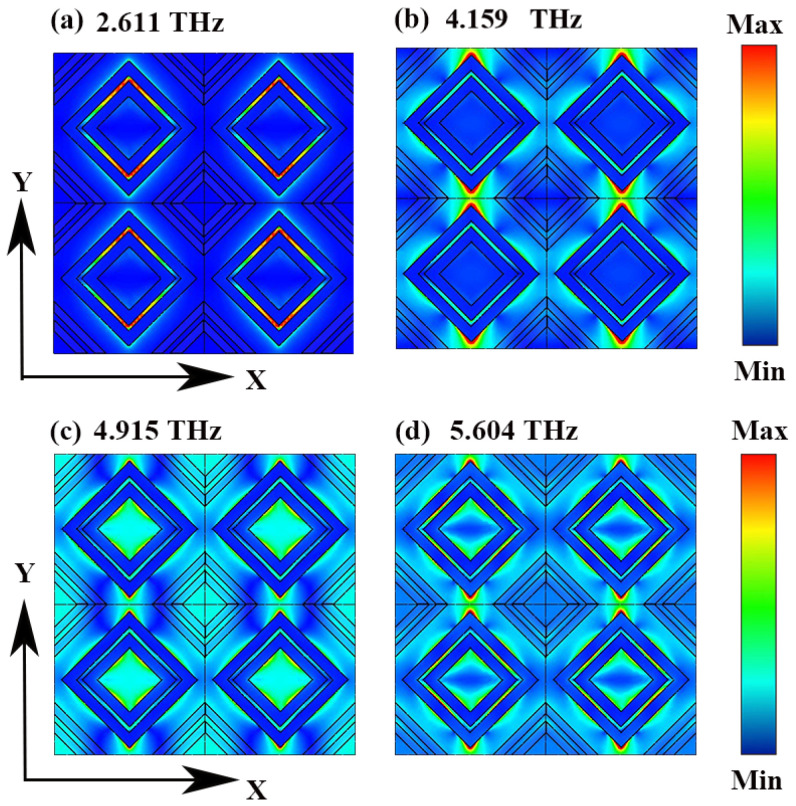
Absolute electric field distributions of DSM at (**a**) 2.611 THz, (**b**) 4.159 THz, (**c**) 4.915, and (**d**) 5.604 THz.

In order to explore the physical mechanisms of the four absorption modes, we analyzed the electric field distribution at their resonant frequencies, as shown in Figure 4. The electric field distribution of M_1_ is significantly different from the other three, mainly distributed at the upper and lower ends of the fillister between the two lozenge rings. The alternative absorption mode involves electric fields distributed around the lozenge ring, which features a broader radius. The field strength distribution is higher in modes M2 and M4 compared to M3, resulting in their enhanced absorptivity. The key difference lies in the distribution of electric fields: M3 exhibits electric fields both around the surrounding trapezoidal structure and within the central groove, whereas M4 has electric fields only within the central groove, and M2 lacks such fields entirely. The localized surface plasmon effect is responsible for the resonances observed at the device’s four absorption peak frequencies, as illustrated in the electric field distribution diagram [39]. This effect induces a strong local electric field enhancement in the surface and near-surface regions of the structure, thereby increasing the local electromagnetic energy density and enhancing the absorption of electromagnetic energy [40]. At these resonant frequencies, the intrinsic losses within the device are maximized, improving its efficiency in converting terahertz (THz) wave energy. As a result, when THz waves interact with the device, their energy is completely absorbed and dissipated as heat, leading to perfect absorption [41].

To further explain the phenomenon of perfect absorption in the device, we used relative impedance matching for analysis. A critical prerequisite for achieving perfect absorption is that the incident frequency of the electromagnetic waves must align with the resonance frequency of the plasmonic constituents. When the incident electromagnetic wave frequency precisely matches the resonance frequency of the plasmonic elements, the absorption effect is maximized [42]. This occurs because the electromagnetic energy is efficiently coupled into the oscillation modes of the plasmonic elements, thereby maximizing energy loss. The effective impedance of the absorber can be represented by the S parameter [43]:

(6) $${ⱬ}_{r}=\pm \sqrt{\frac{{\left(1+S_{11}(\omega )\right)}^{2}-S_{21}^{2}(\omega )}{{\left(1-S_{11}(\omega )\right)}^{2}-S_{21}^{2}(\omega )}}$$

**Figure 5:**
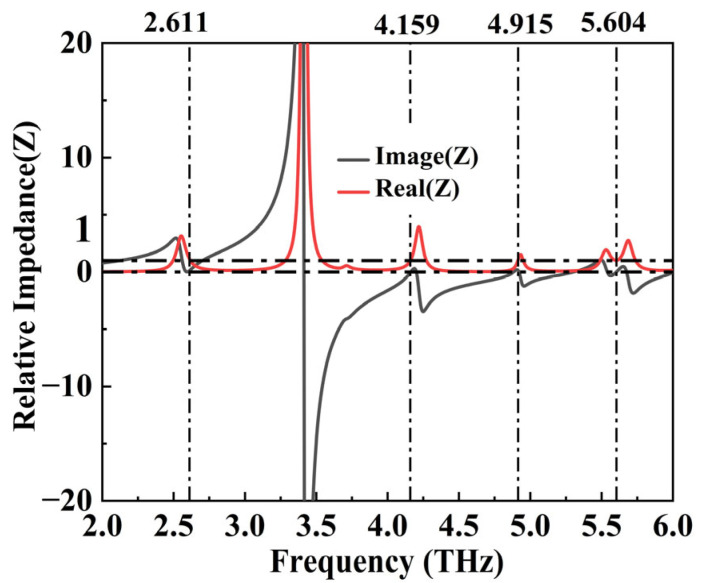
The surface impedance of the absorber (Zr).

Among them, *S*_11_ (ω) and *S*_22_ (ω) are the reflection coefficient and transmission coefficient in the S parameter, respectively. The simulation calculation results show that the virtual impedance of our device at the four absorption peak resonance frequencies is 1.12 + 0.12i, 1.00 − 0.04i, 1.09 + 0.14i, and 1.01 + 0.01i, respectively. The real and imaginary components of the above results are close to 1 and 0, respectively, perfectly matching the free space. In addition, we also plotted the effective impedance diagram of the device in the range of 2.0 THz to 6.0 THz, as shown in Figure 5. It is distinctly evident that the relative impedance of the device matches well with the free space impedance [44].

### 3.3. The Influence of Structural Parameters on Device Performance

**Figure 6:**
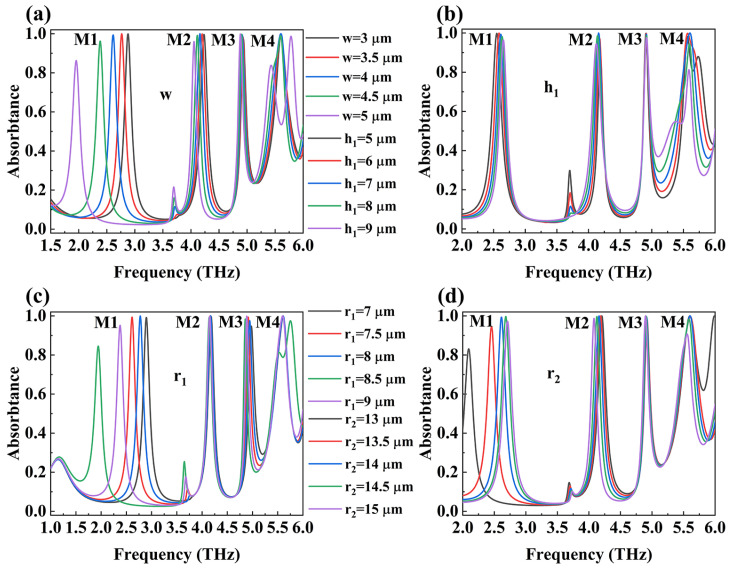
The calculated absorptance spectra of the designed absorber at varying structure parameters (w (part (**a**), h_1_ = 7 μm, r_1_ = 8 μm, r_2_ = 14 μm), h1 (part (**b**), w = 4 μm, r_1_ = 8 μm, r_2_ = 14 μm), r1 (part (**c**), w = 4, h_1_ = 7 μm, r_2_ = 14 μm), and r_2_ (part (**d**), w = 4, h_1_ = 7, r_1_ = 8)) when the conductivity of vanadium dioxide is equal to 250,000 S/m.

After verifying the rationality of perfect absorption in the device, we studied the effects of the relevant structural parameters (w, h_1_, r_1_, and r_2_) in the top DSM absorption layer on absorption. We conducted a sequential study on the relevant parameters, and when analyzing the effect of one parameter, all other parameters remained unchanged. The results are shown in Figure 6. As the parameter w increases, the resonance frequencies of all four absorption peaks exhibit a bathochromic shift, with the greatest shift observed for M1. During this redshift process, the absorption rates of M1 and M2 decrease, while those of M3 and M4 remain relatively constant. However, M4 undergoes splitting at w = 5 μm. The first three absorption peaks are largely unaffected by changes in h1, with M1 and M2 showing increasing and decreasing resonance frequencies, respectively, while maintaining stable absorption rates. M3 shows no significant variation in either absorption rate or frequency. M4 does not shift in frequency but displays a gradual decrease in its absorption rate.

As the parameter r1 increases, the first three absorption peaks exhibit a redshift. M3’s absorption rate initially increases and then decreases, whereas the absorption rates of M1 and M2 decline. M4 experiences a minimal frequency shift, but its absorption peak moves towards a higher resonance frequency when r1 reaches its maximum value. Finally, as r2 increases, M1 undergoes a blue-shift while M2 experiences a redshift; M1’s absorption rate increases initially before decreasing, while M2’s absorption remains relatively stable. M3’s absorption rate and resonance frequency remain largely unchanged. The resonance frequency of M4 stabilizes as its absorption rate decreases.

In the above description, we can use the LC circuit model to explain the reason for the redshift of the absorption peak. At this point, the resonant frequency of the absorption peak is equivalently replaced by the capacitance C and inductance L [45,46].

(7) $$ƒ=\frac{1}{2\pi \sqrt{LC}}$$

Among them, C=εrS/4πkd (*ε* is the dielectric constant, *S* is the facing area of the capacitor plate, *d* is the distance between the capacitor plate, and *k* is the electrostatic constant). When L is invariable, the central resonant frequency of the absorber can be changed by varying the value of C. When the spacing between components in the top structure decreases, the coupling between them increases. At this point, we have completed the study on the effect of some of the structural parameters of DSM on the absorptivity of electromagnetic waves by the device.

### 3.4. Coordination of Devices

**Figure 7:**
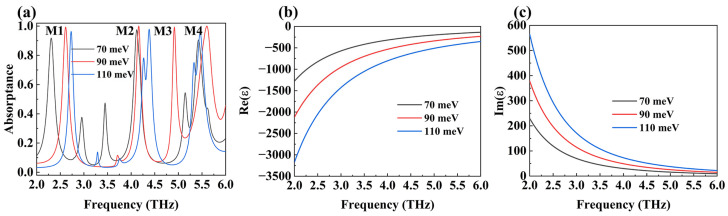
(**a**) The absorption spectra for different E_F_ of DSM. The real (**b**) and the imaginary (**c**) parts of the permittivity for DSM as a function of frequency with the distinct Fermi levels.

As mentioned earlier, DSM is a coordinated material that can change its conductivity by adjusting its Fermi energy. Therefore, our absorber can also regulate the center frequencies of the four absorption peaks by changing the Fermi level of the DSM absorption layer, as shown in Figure 7. Let us first observe the alteration of the center frequency: M1 and M2 both increase; M3 decreases and is fused with M2 at 110 meV. M4 first increases and then decreases. The maximum adjustable frequency ranges for M1, M2, and M4 are 0.288 THz, 0.08 THz, and 0.03 THz, respectively. During the movement of the absorption peak, the absorptivity of the four also changed. M1 and M4 both increase first and then decrease; M2 remains; and M3 increases and merges with M2. Hence, one can see that our device can adjust the resonant frequency of the absorption peak. We can utilize the perturbation theory to explain the reasons for the blue-shift of the four absorption peaks in our device [47,48]:

(8) $$\frac{∆\omega }{\omega_{0}}=\frac{\omega -\omega_{0}}{\omega_{0}}\approx \frac{-\iint_{v}dV\left[\left(∆\vec{\varepsilon }\times \vec{E}\right)\times {\vec{E_{0}}}^{*}+\left(∆\vec{\mu }\times \vec{H}\right)\times {\vec{H_{0}}}^{*}\right]}{∭dV\left(\varepsilon {\left|\vec{E_{0}}\right|}^{2}+\mu {\left|\vec{H_{0}}\right|}^{2}\right)}$$

where ∆*ε* and ∆*µ* represent the changes in the dielectric constant and magnetic permeability of the DSM system, respectively. $\vec{E_{0}}$ and $\vec{H_{0}}$ are the undisturbed electric and magnetic fields, respectively. $\vec{E}$ and $\vec{H}$ are the disturbed electric magnetic fields, separately. ∆ω is the electromagnetic energy change induced by material disturbance, which is proportional to the change in the dielectric constant and the strong light field, as shown in Equation (8). For this reason, the resonance frequency of the absorption peak of the device can be effectively modulated by adjusting the dielectric constant of the material in a strong electric field [49,50]. From Figure 7b, it can be seen that as E_F_ magnifies, the real component of the dielectric constant of the DSM reduces little by little. Both $\vec{E_{0}}$ and $\vec{H_{0}}$ are less than 0; through Equation (8), it can be concluded that an augment in the Fermi level is accompanied by an increase in resonance frequency, resulting in a blue-shift.

**Figure 8:**
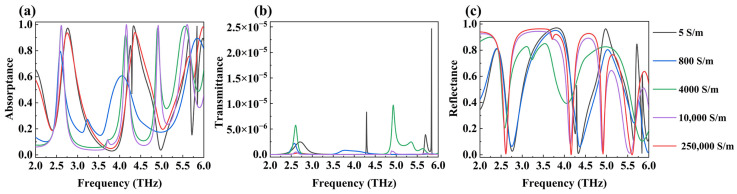
The absorption (**a**), transmission (**b**), and reflection (**c**) spectra of VO_2_ absorbers with different conductivity in the range of 1.5 to 6.0 THz.

In addition to adjusting the center frequency of the absorption peak by changing the Fermi level of DSM, we can also trigger the phase transition of VO_2_, change its conductivity, and thus alter the level of the absorption peak, as shown in Figure 8. In simulation calculations, we simulated the phase transformation of the VO_2_ substrate by adjusting its conductivity. The conductivity of vanadium dioxide was sequentially set to 5 S/m, 800 S/m, 4000 S/m, 10,000 S/m, and 250,000 S/m. We define VO_2_ as a dielectric state when its conductivity is 5 S/m and as a metallic state when its conductivity is 250,000 S/m. When the conductivity of VO_2_ rises (during a phase transition from non-metallic to metallic state), the absorptivity of the equipment gradually improves, while the transmittance and reflectivity gradually decrease. The absorption rates of the four absorption peaks increases from 58.8%, 48.4%, 10.2%, and 80.7% to 99.3%, 99.9%, and 99.9%, respectively. The transmittance and reflectance of the four absorption peaks gradually approach 0. This is because the phase transition process of VO_2_ also involves transformations in the crystal structure. In that case, the number of free electrons inside it multiplies sharply, and the metal’s properties and its reflection ability towards THz are enhanced [51]. While VO_2_ transitions from a non-metallic state to a metallic state, the device forms a stable F-B resonant cavity, which is one of the necessary conditions of perfect absorption [52]. At this time, the DSM absorption layer serves as the front reflector of the resonant cavity, and VO_2_ serves as the bottom reflector, causing critical coupling of the device. Then, the absorption rate of the metamaterial layer is approximately equal to the rate at which electromagnetic waves enter the cavity; the device produces perfect absorption. On the contrary, when we reduced the conductivity of VO_2_ through its phase transition, it weakened the device’s ability to absorb electromagnetic waves. Furthermore, the phase transition of VO_2_ has various advantages, such as controllability and easy triggering. So, our device’s ability to absorb THz waves is adjustable, which is another coordinated usage method of the device.

### 3.5. Polarization Sensitivity of Devices

**Figure 9:**
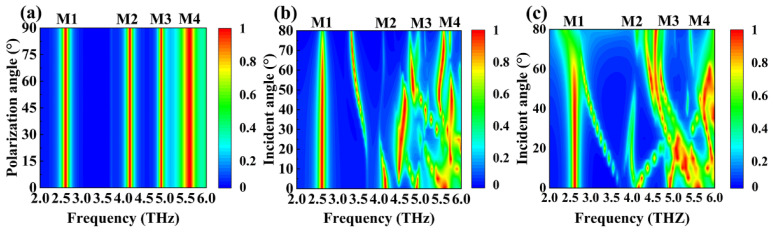
The absorption spectra under different incidence angles for (**a**) the polarization mode, (**b**) the TE mode, and (**c**) the TM mode.

In addition, due to the symmetry of the device structure design, under the condition of THz wave vertical incidence, its absorption of THz waves is not affected by the change of the polarization angle [53,54]. In other words, our absorber posseses both perfect absorption and excellent polarization stability, as shown in Figure 9a. Furthermore, the highly symmetrical shape allows our device to successfully couple incident waves in both TE and TM modes while exhibiting similar absorption properties [55,56]. Figure 9 b, c demonstrate the absorber’s absorption of terahertz waves at various incidence angles in the 2.0 to 6.0 THz range under TE and TM modes, respectively. The direct color changes in blue and red indicate the strength of the absorption ability. As shown in Figure 9b, When the incident angle increases in the TE mode, the situation of M1 is the most unaffected. When the angle of incidence is under 30°, M2 remains stable. When it exceeds 30°, the absorptivity of M2 decreases to about 50%, and a new absorption peak separates in the direction of M1, with its absorptivity over 90%. When the incident angle is less than 10°, M3 stabilizes; when it is greater than 10°, the resonance frequency of M3 shifts to 4.5 THz; when it is greater than 50°, M3 offsets back; and when it exceeds 70°, the absorptivity of M3 decreases to 65%. When the incident angle is less than 60°, M4 remains stable but moves towards a lower resonant frequency when it exceeds 60°. As shown in Figure 9c, in the TM mode, M1 and M4 are more stable than others, approaching perfect absorption when the incident angle is less than 60° and decreasing to 70% when the incident angle is greater than 60°. As the incident angle magnifies, the M2 and M3 peaks gradually disappear, but a new absorption peak appears between them, with an absorption rate of over 95% near 4.8 THz. After the above analysis, we can see that our device not only has excellent polarization stability but also can cope with Hertzian waves incident at various angles in both TE and TM modes.

### 3.6. Sensing Performance

**Figure 10:**
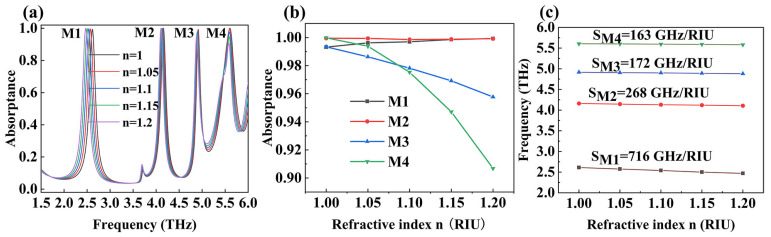
The influence of the environmental refractive index on device absorption, (**a**) changes in the absorption spectrum, (**b**) changes in the absorption index, (**c**) changes in central resonant frequency, and the sensitivity of the environmental refractive index.

In order to further explore the practical application value of the device we proposed, we also analyzed its sensing performance. As shown in Figure 10, we investigated the changes in device absorption performance when the environmental refractive index (n) increased from 1.05 to 1.2 with a step size of 0.05. From Figure 10a, we can simultaneously observe the variations in the absorption rates and the central resonant frequency of the four absorption peaks of the device. Figure 10b and Figure 10c, respectively, show the variations in the absorption rates of the four absorption peaks and the shift of the central resonant frequency as the environmental refractive index magnifies. We can observe that as n magnifies, the absorptivity of M1 gradually increases, from 99.34% to 99.93%. Although the absorption rate of M2 initially decreases, it later returns to its original absorption level. The absorption rates of M3 and M4 gradually decrease, with the latter having a greater amplitude than the former, decreasing from 99.34% and 99.98% to 95.77% and 90.68%, respectively. Meanwhile, as the environmental refractive index gradually magnifies, the resonant frequencies of the four absorption peaks of the device decrease, as shown in Figure 10c. In order to qualitatively analyze the sensitivity of the four absorption peaks of the device to variations in the environmental refractive index, we will use environmental refractive index sensitivity (S) for discussion [57,58,59]. The definition of environmental refractive index sensitivity is the ratio of the change in absorption peak resonance frequency to the change in the environmental refractive index, measured in GHz/RIU. The calculation formula is as follows [60,61,62,63]:

(9) $$S=\frac{∆f}{∆n}$$

Among them, ∆f and ∆n represent the changes in absorption peak resonance frequency and the environmental refractive index, respectively. The calculation results of the sensitivity of the environmental refractive index for the four absorption peaks are as follows: $S_{M1}=716\;GHz/RIU$, $S_{M2}=268\;GHz/RIU$, $S_{M3}=172\;GHz/RIU$, and $S_{M4}=163\;GHz/RIU$. The calculation results show that the THz metamaterial absorber we designed has extremely high environmental refractive index sensitivity and is very sensitive to changes in the refractive index in the working environment, which allows it the potential certain applications in fields such as biosensors and medical diagnosis. Finally, we will compare the performance indicators of the terahertz metamaterial absorber designed in this article with some similar absorbers currently in the market, as shown in Table 1 [64,65,66,67,68,69,70,71,72,73]. The results show that the absorber we designed has a simpler structure, lower production costs, and stronger functionality.

## 4. Conclusions

In this research, we designed a THz metamaterial absorber with two coordinated modes utilizing two suitable materials: Dirac semimetal and vanadium dioxide. When VO_2_ is in the metallic form, the device has four distinct absorption modes (M1, M2, M3, and M4) in the frequency range of 2.0 THz to 6.0 THz, with absorption rates of 99.3%, 99.9%, 99.3%, and 99.9%, all of which are greater than 99%. When VO_2_ is in a non-metallic form, the four absorption peaks absorb at rates of 58.8%, 48.4%, 10.2%, and 80.7%. Furthermore, the central resonance frequencies of these absorption peaks can be dynamically tuned by adjusting the Fermi energy of the DSM layer through electrostatic doping, achieving a maximum tuning range of 0.288 THz. We explained the physical mechanism of perfect absorption in the device from two perspectives: relative impedance matching of the device and analysis of the electric field distribution. At the same time, we studied the influence of some structural parameters of the device on absorption. Owing to the highly symmetrical structure of our absorber, it demonstrates excellent polarization insensitivity and can accommodate wide-angle incidence for both transverse electric (TE) and transverse magnetic (TM) modes. Moreover, the device exhibits high sensitivity to variations in the surrounding refractive index, achieving up to 716 GHz per refractive index unit (RIU). In summary, the proposed terahertz absorber features multiple tuning mechanisms for modulation and is capable of absorbing multiple specific frequencies with minimal interference. Coupled with key characteristics such as polarization insensitivity and polarization stability, it can become an important component of modern THz communication systems, especially in high-speed data transmission and secure communication fields.

## Figures and Tables

**Table 1:** Performance comparison of different absorbers.

Critical Material	Number of Layers	Absorption Type	Absorption Rate	Tunable Range	Ref.
Aluminum	3	One-band absorption	99%	No research	[64]
Vanadium dioxide	4	Broadband absorption	99.9%	No research	[65]
Aluminum	3	Three-band absorption	70%	0.005 THz	[66]
Graphene	12	Three-band absorption	95.3%	0.046 THz	[67]
Graphene	2	One-band absorption	93%	2.2 THz	[68]
Graphene	6	Two-band absorption	97.4%	0.5 THz	[69]
Graphene	4	Three-band absorption	99%	0.15 THz	[70]
Graphene and … Vanadium dioxide	3	Broadband absorption	97.4%	1 THz/0%~97.4%	[71]
Vanadium dioxide	3	Broadband absorption	99.9%	4~100%	[72]
Graphene	3	Two-band absorption	80%	0.5 THz	[73]
Dirac semimetals and Vanadium dioxide	3	Four-band absorption	99.9%	0.288 THz/10%~99.3%	This paper

## Data Availability

Publicly available datasets were analyzed in this study. These data can be found here: [https://www.lumerical.com/, accessed on 10 October 2024].
